# The impact of text segmentation on subtitle reading

**DOI:** 10.16910/jemr.11.4.2

**Published:** 2018-06-30

**Authors:** Olivia Gerber-Morón, Agnieszka Szarkowska, Bencie Woll

**Affiliations:** Universitat Autònoma de Barcelona, Spain; University College London, UK; University of Warsaw, Poland

**Keywords:** eye movement, reading, region of interest, subtitling, audiovisual translation, media accessibility, cognitive load, segmentation, line breaks, revisits

## Abstract

Understanding the way people watch subtitled films has become a central concern for subtitling researchers in recent years. Both subtitling scholars and professionals generally believe that in order to reduce cognitive load and enhance readability, line breaks in twoline subtitles should follow syntactic units. However, previous research has been inconclusive as to whether syntactic-based segmentation facilitates comprehension and reduces cognitive load. In this study, we assessed the impact of text segmentation on subtitle processing among different groups of viewers: hearing people with different mother tongues (English, Polish, and Spanish) and deaf, hard of hearing, and hearing people with English as a first language. We measured three indicators of cognitive load (difficulty, effort, and frustration) as well as comprehension and eye tracking variables. Participants watched two video excerpts with syntactically and non-syntactically segmented subtitles. The aim was to determine whether syntactic-based text segmentation as well as the viewers’ linguistic background influence subtitle processing. Our findings show that non-syntactically segmented subtitles induced higher cognitive load, but they did not adversely affect comprehension. The results are discussed in the context of cognitive load, audiovisual translation, and deafness.

## Introduction 

In the modern world, we are surrounded by screens, captions, and moving images more than ever before. Technological advancements and accessibility legislation, such as the United Nations Convention on the Rights of Persons with Disabilities (2006), Audiovisual Media Services Directive or the European Accessibility Act,have empowered different types of viewers across the globe in accessing multilingual audiovisual content. Viewers who do not know the language of the original production or people who are deaf or hard of hearing can follow film dialogues thanks to subtitles (15).

Because watching subtitled films requires viewers to follow the action, listen to the soundtrack and read the subtitles, it is important for subtitles to be presented in a way that facilitates rather than hampers reading (12, 21). Some typographical subtitle parameters, such as small font size, illegible typeface or optical blur, have been shown to impede reading (2, 63). In this study, we examine whether segmentation, i.e. the way text is divided across lines in a two-line subtitle, affects the subtitle reading process. We predict that segmentation not aligned with grammatical structure may have a detrimental effect on the processing of subtitles.

### Readability and syntactic segmentation in subtitles

The general consensus among scholars in audiovisual translation, media regulation, and television broadcasting is that to enhance readability, linguistic phrases in two-line subtitles should not be split across lines (6, 12, 18, 21, 42). For instance, subtitle (1a) below is an example of correct syntactic-based line segmentation, whereas in (1b) the indefinite article “a” is incorrectly separated from the accompanying noun phrase (6).

(1a)

We are aiming to get

a better television service.

(1b)

We are aiming to get a

better television service.

The underlying assumption is that more cognitive effort is required to process text when it is not segmented according to syntactic rules (44). However, segmentation rules are not always respected in the subtitling industry. One of the reasons for this might be the cost: editing text in subtitles requires human time and effort, and as such is not always cost-effective. Another reason is that syntactic-based segmentation may require substantial text reduction in order to comply with maximum line length limits. As a result, when applying syntactic rules to segmentation of subtitles, some information might be lost. Following this line of thought, BBC subtitling guidelines (6) stress that well-edited text and synchronisation should be prioritized over syntactically-based line breaks.

The widely held belief that words “intimately connected by logic, semantics, or grammar” should be kept in the same line whenever possible (18, p. 77) may be rooted in the concept of parsing in reading (51, p. 216). Parsing, i.e. the process of identifying which groups of words go together in a sentence (68), allows a text to be interpreted incrementally as it is read. It has been reported that “line breaks, like punctuation, may have quite profound effects on the reader’s segmentation strategies” (23, p. 56). Insight into these strategies can be obtained through studies of readers’ eye movements, which reflect the process of parsing: longer fixation durations, higher frequency of regressions, and longer reading time may be indicative of processing difficulties (49). An inappropriately placed line break may lead a reader to incorrectly interpret the meaning and structure, luring the reader into a parse that turns out to be a dead end or yield a clearly unintended reading – a so-called “garden path” experience (14, 51). The reader must then reject their initial interpretation and re-read the text. This takes extra time and, as such, is unwanted in subtitling, which is supposed to be as unobtrusive as possible and should not interfere with the viewer’s enjoyment of the moving images (12).

Despite a substantial body of experimental research on subtitling (7, 9, 10, 24, 29, 30, 47, 61), the question of whether text segmentation affects subtitle processing (44) still remains unanswered. Previous research is inconclusive as to whether linguistically segmented text facilitates subtitle processing and comprehension. Contrary to arguments underpinning professional subtitling recommendations, Perego, Del Missier, Porta, & Mosconi (46), who used eye-tracking to examine subtitle comprehension and processing, found no disruptive effect of “syntactically incoherent” segmentation of noun phrases on the effectiveness of subtitle processing in Italian. In their study, the number of fixations and saccadic crossovers (i.e. gaze jumps between the image and the subtitle) did not differ between the syntactically segmented and non-segmented conditions. In contrast, in a study on live subtitling, Rajendran, Duchowski, Orero, Martínez, & Romero-Fresco (48) showed benefits of linguistically-based segmentation by phrase, which induced fewer fixations and saccadic crossovers, and resulted in shortest mean fixation duration, together indicating less effortful processing.

Ivarsson & Carroll (18) noted that “matching line breaks with sense blocks is especially important for viewers with any kind of linguistic disadvantage, e.g. immigrants or young children learning to read or the deaf with their acknowledged reading problems” (p. 78). Indeed, early deafness is strongly associated with reading difficulties (37, 41). Researchers investigating subtitle reading by deaf viewers have demonstrated processing difficulties resulting in lower comprehension and more time spent by deaf viewers on reading subtitles (25, 26, 60). Lack of familiarity with subtitling is another aspect which may affect the way people read subtitles. In a recent study, Perego et al. (47) found that subtitling can hinder viewers accustomed to dubbing from fully processing film images, especially in the case of structurally complex subtitles.

### Cognitive load

Watching a subtitled video is a complex task: not only do viewers need to follow the dynamically unfolding on-screen actions, accompanied by various sounds, but they also need to read the subtitles (32). This complex processing task may be hindered by poor quality subtitles, possibly including aspects such as non-syntactic segmentation. The processing of subtitles has been previously studied in association with the concept of cognitive load (27), rooted in cognitive load theory (CLT) and instructional design (56). Drawing on the central tenet of CLT, the design of materials should aim at reducing any unnecessary load to free the processing capacity for task-related activities (58).

In the initial formulation of CLT, two types of cognitive load were distinguished: intrinsic and extraneous (8). Intrinsic cognitive load is related to the complexity and characteristics of the task (54). Extraneous load relates to how the information is presented; if presentation is inefficient, learning can be hindered (57). For instance, too many colours or blinking headlines in a lecture presentation can distract students rather than help them focus, wasting attentional resources on taskirrelevant details (54). Later studies in CLT also distinguish the concept of ‘germane cognitive load’ and, more recently, ‘germane resources’ (54, 57). It is believed that germane load is not imposed by the characteristics of the materials and germane resources should be “high enough to deal with the intrinsic cognitive load caused by the content” (54). In this paper, we set out to test whether non-syntactically segmented text may strain working memory capacity and prevent viewers from efficiently processing subtitled videos. It is our contention that just as the goal of instructional designers is to foster learning by keeping extraneous cognitive load as low as possible (54), so it is the task of subtitlers to reduce the extraneous load on viewers, enabling them to focus on what is important during the filmwatching experience.

The concept of cognitive load encompasses different categories (58, 67). Mental effort is understood, following Paas, Tuovinen, Tabbers, & Van Gerven (43, p. 64) and Sweller et al. (57, p. 73), as “the aspect of cognitive load that refers to the cognitive capacity that is actually allocated to accommodate the demands imposed by the task”. As mental effort invested in a task is not necessarily equal to the difficulty of the task, difficulty is a construct distinct from effort (66). Drawing on the multidimensional NASA Task Load Index (16), some researchers also included other aspects of cognitive load, such as temporal demand, performance, and frustration with the task (57). Apart from effort, difficulty and frustration, of particular importance in the present study is performance, operationalised here as comprehension score, which demonstrates how well a person carried out the task. Performance may be positively affected by lower cognitive load, as there is more unallocated processing capacity to carry out the task. As the task complexity increases, more effort needs to be expended to keep the performance at the same level (43).

Cognitive load can be measured using subjective or objective methods (27, 57). Subjective cognitive load measurement is usually done indirectly using rating scales (43, 54), where people are asked to rate their mental effort or the perceived difficulty of a task on a 7- or 9-point Likert scale, ranging from “very low” to “very high” (66). Subjective rating scales have been criticised for using only one single item (usually either mental load or difficulty) in assessing cognitive load (54). Yet, they have been found to effectively show the correlations between the variation in cognitive load reported by people and the variation in the complexity of the task they were given (43). According to Sweller et al. (57), “the simple subjective rating scale [...], has, perhaps surprisingly, been shown to be the most sensitive measure available to differentiate the cognitive load imposed by different instructional procedures” (p. 74). The problem with rating scales is they are applied to the task as a whole, after it has been completed. In contrast, objective methods, which include physiological tools such as eye tracking or electroencephalography (EEG), enable researchers to see fluctuations in cognitive load over time (3, 65). Higher number of fixations and longer fixation durations are generally associated with higher processing effort and increased cognitive load (17, 28). In our study, we combine subjective rating scales with objective eye-tracking measures to obtain a more reliable view on cognitive load during the task of subtitle processing.

Various types of measures have been used to evaluate cognitive load in subtitling. Some previous studies have used subjective post-hoc rating scales to assess people’s cognitive load when watching subtitled audiovisual material (27, 30, 69); subtitlers’ cognitive load when producing live subtitles with respeaking (62); or the level of translation difficulty (55). Some studies on subtitling have used eye tracking to examine cognitive load and attention distribution in a subtitled lecture (30); cognitive load while reading edited and verbatim subtitles (60); or the processing of native and foreign subtitles in films (7); to mention just a few. Using both eye tracking and subjective self-report ratings, Łuczak (34) tested the impact of the language of the soundtrack (English, Hungarian, or no audio) on viewers’ cognitive load. Kruger, Doherty, Fox, et al. (28) combined eye tracking, EEG and selfreported psychometrics in their examination of the effects of language and subtitle placement on cognitive load in traditional intralingual subtitling and experimental integrated titles. For a critical overview of eye tracking measures used in empirical research on subtitling, see (13), and of the applications of cognitive load theory to subtitling research, see Kruger & Doherty (27).

### Overview of the current study

The main goal of this study is to test the impact of segmentation on subtitle processing. With this goal in mind, we showed participants two videos: one with syntactically segmented text in the subtitles (SS) and one where text was not syntactically segmented (NSS). In order to compensate for any differences in the knowledge of source language and accessibility of the soundtrack to deaf and hearing participants, we used videos where the soundtrack was in Hungarian – a language that participants could not understand.

All subtitles in this study were shown in English. The reason for this is threefold. First, the noncompliance with the subtitling guidelines with regard to text segmentation and line breaks is particularly visible on British television in Englishto-English subtitling. Although the UK is the leader in subtitling when it comes to the quantity of subtitle provision, with many TV channels having 100% subtitling to its programmes, the quality of prerecorded subtitles is often below professional subtitling standards with regard to subtitle segmentation. Another reason for using English – as opposed to showing participants subtitles in their respective mother tongues – was to ensure identical linguistic structures in the subtitles. A final reason for using English is that, as participants live in the UK, they are able to watch English subtitles on television. The choice of English subtitles is therefore ecologically valid.

We measured participants’ cognitive load and comprehension as well as a number of eye tracking variables. Following the established method of measuring self-reported cognitive load previously used by Kruger et al. (30), (61), and Łuczak (34), we measured three aspects of cognitive load: perceived difficulty, effort, and frustration, using subjective 17 rating scales (54). We also related viewers’ cognitive load to their performance, operationalised here as comprehension score. Based on the subtitling literature (45), we predicted that non-syntactically segmented text in subtitles would result in higher cognitive load and lower comprehension. We hypothesised that subtitles in the NSS condition would be more difficult to read because of increased parsing difficulties and extra cognitive resources which might be expended on additional processing.

In terms of eye tracking, we hypothesised that people would spend more time reading subtitles in the NSS condition. To measure this, we calculated the absolute reading time and proportional reading time of subtitles as well as fixation count in the subtitles. Absolute reading time is the time the viewers spent in the subtitle area, measured in milliseconds, whereas proportional reading time is a percentage of time spent in the subtitle area relative to subtitle duration (11, 24). Furthermore, because we thought that the non-syntactically segmented text would be more difficult to process, we also expected higher mean fixation duration and more revisits to the subtitle area in the NSS condition (17, 50, 51).

To address the contribution of hearing status and experience with subtitling to cognitive processing, our study includes British viewers with varying hearing status (deaf, hard of hearing, and hearing), and hearing native speakers of different languages: Spanish people, who grew up in a country where the dominant type of audiovisual translation is dubbing, and Polish people, who come from the tradition of voice-over and subtitling. We conducted two experiments: Experiment 1 with hearing people from the UK, Poland, and Spain, and Experiment 2 with English hearing, hard of hearing and deaf people. We predicted that for those who are not used to subtitling, cognitive load would be higher, comprehension would be lower and time spent in the subtitle would be higher, as indicated by absolute reading time, fixation count and proportional reading time.

By using a combination of different research methods, such as eye tracking, self-reports, and questionnaires, we have been able to analyse the impact of text segmentation on the processing of subtitles, modulated by different linguistic backgrounds of viewers. Examining these issues is particularly relevant from the point of view of current subtitling standards and practices.

## Methods

The study took place at University College London and was part of a larger project on testing subtitle processing with eye tracking. In this paper, we report the results from two experiments using the same methodology and materials: Experiment 1 with hearing native speakers of English, Polish, and Spanish; and Experiment 2 with hearing, hard of hearing, and deaf British participants. The Englishspeaking hearing participants are the same in both experiments. In each of the two experiments, we employed a mixed factorial design with segmentation (syntactically segmented vs. nonsyntactically segmented) as the main within-subject independent variable, and language (Exp. 1) or hearing loss (Exp. 2) as a between-subject factor.

All the study materials and results are available in an open data repository RepOD hosted by the University of Warsaw (59).

### Participants 

Participants were recruited from the UCL Psychology pool of volunteers, social media (Facebook page of the project, Twitter), and personal networking. Hard of hearing participants were recruited with the help of the National Association of Deafened People. Deaf participants were also contacted through the UCL Deafness, Cognition, and Language Research Centre participant pool. Participants were required not to know Hungarian.

**Table 1.** Demographic information on participants

Experiment 1				
		English	Polish	Spanish
Gender	Male	13	5	10
	Female	14	16	16
Age	Mean	27.59	24.71	28.12
	(SD)	(7.79)	(5.68)	(5.88)
	Range	20-54	19-38	19-42
Experiment 2				
		Hearing	Hard of hearing	Deaf
Gender	Male	13	2	4
	Female	14	8	5
Age	Mean	27.59	46.40	42.33
	(SD)	(7.79)	(12.9)	(14.18)
	Range	20-54	22-72	24-74

Experiment 1 participants were pre-screened to be native speakers of English, Polish or Spanish, aged above 18. They were all resident in the UK. We tested 27 English, 21 Polish, and 26 Spanish speakers (see Table 1). At the study planning and design stage, Spanish speakers were included on the assumption that they would be unaccustomed to subtitling as they come from Spain, a country in which foreign programming is traditionally presented with dubbing. Polish participants were included as Poland is a country where voice-over and subtitling are commonly used, the former on television and VOD, and the latter in cinemas, DVDs, and VOD. The hearing English participants were used as a control group.

Despite their experiences in their native countries, when asked about the preferred type of audiovisual translation (AVT), most of the Spanish participants declared they preferred subtitling and many of the Polish participants reported that they watch films in the original (see Table 2).

**Table 2.** Preferred way of watching foreign films

	English	Polish	Spanish
Subtitling	24	11	22
Dubbing	0	0	1
Voice-over	1	0	0
I watch films in their original version	1	10	3
I never watch foreign films	1	0	0

**Figure 1.**
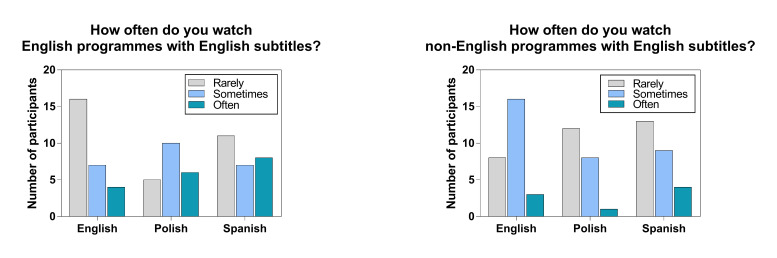
Participants’ subtitle viewing habits

We also asked the participants how often they watched English and non-English programmes with English subtitles (Fig. 1).

The heterogeneity of participants’ habits and preferences reflects the changing AVT landscape in Europe (36) on the one hand, and on the other, may be attributed to the fact that participants were living in the UK and thus had different experiences of audiovisual translation than in their home countries. The participants’ profiles make them not fully representative of the Spanish/Polish population, which we acknowledge here as a limitation of the study.

To determine the level of participants’ education, hearing people were asked to state the highest level of education they completed (Table 3, see also Table 5 for hard of hearing and deaf participants). Overall, the sample was relatively well-educated.

**Table 3.** Education background of hearing participants in Experiment 1

	English	Polish	Spanish
Secondary education	5	9	6
Bachelor degree	14	4	6
Master degree	8	8	13
PhD	0	0	1

As subtitles used in the experiments were in English, we asked Polish and Spanish speakers to assess their proficiency in reading English using the Common European Framework of Reference for Languages (from A1 to C2), see Table 4. None of the participants declared a reading level lower than B1. The difference between the proficiency in English of Polish and Spanish participants was not statistically significant, *χ^2^*(3) = 5.144, *p* = .162. Before declaring their proficiency, each participant was presented with a sheet describing the skills and competences required at each proficiency level (59). There is evidence that self-report correlates reasonably well with objective assessments (35).

**Table 4.** Self-reported English proficiency in reading of Polish and Spanish participants

	Polish	Spanish
B1	0	1
B2	0	4
C1	3	5
C2	18	16
Total	21	26

In Experiment 2, participants were classified as either hearing, hard of hearing, or deaf. Before taking part in the study, those with hearing impairment completed a questionnaire about the severity of their hearing impairment, age of onset of hearing impairment, communication preferences, etc. and were asked if they described themselves as deaf or hard of hearing. They were also asked to indicate their education background (see Table 5). We recruited 27 hearing, 10 hard of hearing, and 9 deaf participants. Of the deaf and hard of hearing participants, 7 were born deaf or hard of hearing, 4 lost hearing under the age of 8, 2 lost hearing between the ages of 9-17, and 6 lost hearing between the ages of 18-40. Nine were profoundly deaf, 6 were severely deaf, and 4 had a moderate hearing loss. Seventeen of the deaf and hard of hearing participants preferred to use spoken English as their means of communication in the study and two chose to use a British Sign Language interpreter. In relation to AVT, 84.2% stated that they often watch films in English with English subtitles; 78.9% declared they could not follow a film without subtitles; 58% stated that they always or very often watch non-English films with English subtitles. Overall, deaf and hard of hearing participants in our study were experienced subtitle users, who rely on subtitles to follow audiovisual materials.

**Table 5.** Education background of deaf and hard of hearing participants

	Deaf	Hard of hearing
GCSE/O-levels	3	1
A-levels	2	4
University level	4	5

In line with UCL hourly rates for experimental participants, hearing participants received £10 for their participation in the experiment. In recognition of the greater difficulty in recruiting special populations, hard of hearing and deaf participants were paid £25. Travel expenses were reimbursed as required.

### Materials 

These comprised two self-contained 1-minute scenes from films featuring two people engaged in a conversation: one from *Philomena* (Desplat & Frears, 2013) and one from *Chef* (Bespalov & Favreau, 2014). The clips were dubbed into Hungarian – a language unknown to any of the participants and linguistically unrelated to their native languages. Subtitles were displayed in English, while the audio of the films was in Hungarian. Table 6 shows the number of linguistic units manipulated for each clip.

**Table 6.** Number of instances manipulated for each type of linguistic unit

Linguistic unit	Chef	Philomena
Auxiliary and lexical verb	2	2
Subject and predicate	3	3
Article and noun	3	3
Conjunction between two clauses	4	5

Subtitles were prepared in two versions: syntactically segmented and non-syntactically segmented (see Table 7) (SS and NSS, respectively). The SS condition was prepared in accordance with professional subtitling standards, with linguistic phrases appearing on a single line. In the NSS version, syntactic phrases were split between the first and the second line of the subtitle. Both the SS and the NSS versions had identical time codes and contained exactly the same text. The clip from Philomena contained 16 subtitles, of which 13 were manipulated for the purposes of the experiment; Chef contained 22 subtitles, of which 12 were manipulated. Four types of linguistic units were manipulated in the NSS version of both clips (see Tables 6 and 7).

Each participant watched two clips: one from *Philomena* and one from *Chef*; one in the SS and one in the NSS condition. The conditions were counterbalanced and their order of presentation was randomised using SMI Experiment Centre (see 59).

**Table 7.** Examples of line breaks in the SS and the NSS condition

Linguistic unit	SS condition	NSS condition
Auxiliary and lexical verb	Now, should we have served	Now, should we have
	that sandwich?	served that sandwich?
Subject and predicate	That's my son. Get back in there.	That's my son. Get back in there. We
	We got some hungry people.	got some hungry people.
Article and noun	I've loved the hotels,	I've loved the
	the food and everything,	hotels,the food and everything,
Conjunction between two clauses	Now I've made a decision	Now I've made a decision and
	and my mind's made up.	my mind's made up.

### Eye tracking recording 

An SMI RED 250 mobile eye tracker was used in the experiment. Participants’ eye movements were recorded with a sampling rate of 250Hz. The experiment was designed and conducted with the SMI software package Experiment Suite, using the velocity-based saccade detection algorithm. The minimum duration of a fixation was 80ms. The analyses used SMI BeGaze and SPSS v. 24. Eighteen participants whose tracking ratio was below 80% were excluded from the eye tracking analyses (but not from comprehension or cognitive load assessments).

### Dependent variables 

The dependent variables were: 3 indicators of cognitive load (difficulty, effort and frustration), comprehension score, and 5 eye tracking measures.

The following three indicators of cognitive load were measured using self-reports on a 1-7 scale: difficulty (“Was it difficult for you to read the subtitles in this clip?”, ranging from “very easy” to “very difficult”), effort (“Did you have to put a lot of effort into reading the subtitles in this clip?”, ranging from “very little effort” to “a lot of effort”), and frustration (“Did you feel annoyed when reading the subtitles in this clip?”, ranging from “not annoyed at all” to “very annoyed”).

Comprehension was measured as the number of correct answers to a set of five questions per clip about the content, focussing on the information from the dialogue (not the visual elements). See Szarkowska & Gerber-Morón (592018) for the details, including the exact formulations of the questions.

Table 8 contains a description of the eye tracking measures. We drew individual areas of interest (AOIs) on each subtitle in each clip. All eye tracking data reported here comes from AOIs on subtitles

**Table 8.** Description of the eye tracking measures

Eye tracking measure	Description
Absolute reading time	The sum of all fixation durations and saccade durations, starting from the duration of the saccade entering the AOI, referred to in SMI software as ‘glance duration’. Longer time spent on reading may be indicative of difficulties with extracting information (17).
Proportional reading time	The percentage of dwell time (the sum of durations of all fixations and saccades in an AOI starting with the first fixation) a participant spent in the AOI as a function of subtitle display time. For example, if a subtitle lasted for 3 seconds and the participant spent 2.5 seconds in that subtitle, the proportional reading time was 2500/3000 ms = 83% (i.e. while the subtitle was displayed for 3 seconds, the participant was looking at that subtitle for 83% of the time). Longer proportional time spent in the AOI translates into less time available to follow on-screen action.
Mean fixation duration	The duration of a fixation in a subtitle AOI, averaged per clip per participant. Longer mean fixation duration may indicate more effortful cognitive processing (17).
Fixation count	The number of fixations in the AOI, averaged per clip per participant. Higher numbers of fixations have been reported in poor readers (17).
Revisits	The number of glances a participant made to the subtitle AOI after visiting the subtitle for the first time. Revisits to the AOI may indicate problems with processing, as people go back to the AOI to re-read the text.

### Procedure 

The study received full ethical approval from the UCL Research Ethics Committee. Participants were tested individually. They were informed they would take part in an eye tracking study on the quality of subtitles. The details of the experiment were not revealed until the debrief.

After reading the information sheet and signing the informed consent form, each participant underwent a 9-point calibration procedure. There was a training session, whose results were not recorded. Its aim was to familiarise the participants with the experimental procedure and the type of questions that would be asked in the experiment (comprehension and cognitive load). Participants watched the clips with the sound on. After the test, participants’ views on subtitle segmentation were elicited in a brief interview.

Each experiment lasted approx. 90 minutes (including other tests not reported in this paper), depending on the time it took the participants to answer the questions and participate in the interview. Deaf participants had the option of either communicating via a British Sign Language interpreter or by using their preferred combination of spoken language, writing and lip-reading.

## Results 

### Experiment 1

Seventy-four participants took part in this experiment: 27 English, 21 Polish, 26 Spanish.

### Cognitive load 

To examine whether subtitle segmentation affects viewers’ cognitive load, we conducted a 2 x 3 mixed ANOVA on three indicators of cognitive load: difficulty, effort, and frustration, with segmentation as a within-subject independent variable (SS vs. NSS) and language (English, Polish, Spanish) as a between-subject factor. We found a main effect of segmentation on all three aspects of cognitive load, which were consistently higher in the NSS condition compared to the SS one (Table 9).

**Table 9.** Mean cognitive load indicators for different participant groups in Experiment 1

	Language						
	English	Polish	Spanish	df	*F*	*P*	𝜂_p_²
Difficulty				1,71	15,584	< .001*	.18
SS	2.37 (1.27)	2.05 (1.02)	1.96 (1.14)				
NSS	2.63 (1.44)	2.67 (1.46)	3.42 (1.65)				
Effort				1,71	7,788	.007*	.099
SS	2.78 (1.55)	1.90 (1.26)	2.23 (1.50)				
NSS	2.89 (1.60)	2.43 (1.16)	3.54 (2.10)				
Frustration				1,71	27,030	< .001*	.276
SS	2.15 (1.40)	1.38 (.80)	1.62 (.89)				
NSS	3.04 (1.85)	2.48 (1.91)	3.27 (2.07)				

We also found an interaction between segmentation and language in the case of difficulty, *F*(2,71) = 3,494, *p* = .036, 𝜂_p_² = .090, which we separated with simple effects analyses (post-hoc tests with Bonferroni correction). We found a significant main effect of segmentation on the difficulty of reading subtitles among Spanish participants, *F*(1,25) = 19,161, *p*< .001, 𝜂_p_² = .434. Segmentation did not have a statistically significant effect on the difficulty experienced by English participants, *F*(1,26) = ,855, *p* = .364, 𝜂_p_² = .032 or by Polish participants, *F*(1,20) = 2,147, *p* = .158, 𝜂_p_² = .097. To recap, although cognitive load difficulty was declared to be higher by all participants in the NSS condition, only in the case of Spanish participants was the main effect of segmentation statistically significant.

We did not find any significant main effect of language on cognitive load (Table 10), which means that participants reported similar scores regardless of their linguistic background.

**Table 10.** Between-subjects results for cognitive load

Measure	df	*F*	*p*	𝜂_p_²
Difficulty	2,71	.592	.556	.016
Effort	2,71	2.382	.100	.063
Frustration	2,71	1.850	.165	.050

### Comprehension 

To see whether segmentation affects viewers’ performance, we conducted a 2 x 3 mixed ANOVA on segmentation (SS vs. NSS condition) with language (English, Polish, Spanish) as a betweensubject factor. The dependent variable was comprehension score. There was no main effect of segmentation on comprehension *F*(1,71) = .412, *p* = .523, 𝜂_p_² = .006. Table 11 shows descriptive statistics for this analysis. There were no significant interactions.

**Table 11.** Descriptive statistics for comprehension

	Language	Mean	(SD)
Comprehension SS	English	4.11	(1.01)
	Polish	4.48	(.81)
	Spanish	4.08	(1.09)
	Total	4.20	(.99)
Comprehension NSS	English	4.26	(1.02)
	Polish	4.76	(.43)
	Spanish	3.88	(1.21)
	Total	4.27	(1.02)

We found a main effect of language on comprehension, *F*(2,71) = 3,563, *p* = .034, 𝜂_p_² = .091. Pairwise comparisons with Bonferroni correction showed that Polish participants had significantly higher comprehension than Spanish participants, *p* = .031, 95% CI [.05, 1.23]. There was no difference between Polish and English, *p* =.224, 95% CI [-.15, 1.02], or Spanish and English participants, *p* =1.00, 95% CI [-.76, .35].

### Eye tracking measures 

Because of data quality issues, for eye tracking analyses we had to exclude 8 participants from the original sample, leaving 22 English, 19 Polish, and 25 Spanish participants. We found a significant main effect of segmentation on revisits to the subtitle area (Table 12). Participants went back to the subtitles more in the NSS condition (*M_NSS_* = .37, *SD* = .25) compared to the SS one (*M_SS_* = .25, *SD* = .22), implying potential parsing problems. There was no effect of segmentation for any other eye tracking measure (Table 12). There were no interactions.

**Table 12.** Mean eye tracking measures by segmentation in Experiment 1

	Language						
	English	Polish	Spanish	df	*F*	p	𝜂_p_²
Absolute reading time (ms)				1,63	2.950	.091	.045
SS	1614	1634	1856				
NSS	1617	1529	1817				
Proportional reading time				1,63	2.128	.150	.033
SS	.65	.67	.76				
NSS	.66	.62	.74				
Mean fixation duration (ms)				1,63	2.128	.906	.000
SS	209	194	214				
NSS	211	187	218				
Fixation count				1,63	2.279	.136	.035
SS	6.41	6.68	7.27				
NSS	6.45	6.42	6.95				
Revisits				1,63	11.839	.001*	.158
SS	.28	.27	.21				
NSS	.39	.34	.36				

In relation to the between-subject factor, we found a main effect of language on absolute reading time, proportional reading time, mean fixation duration, and fixation count, but not on revisits (see Table 13).

Post-hoc Bonferroni analyses showed that Spanish participants spent significantly more time in the subtitle area compared to English and Polish participants. This was shown by significantly longer absolute reading time in the case of Spanish participants compared to English, *p* = .027, 95% CI [19.20, 422.73], and Polish participants, *p* = .012, 95% CI [44.61, 464.75]. Polish and English participants did not differ from each other in absolute reading time, *p* =1.00, 95% CI [-249.88, 182.45]. There was a tendency approaching significance for fixation count to be higher among Spanish participants than English participants, *p* = .077, 95% CI [-.05, 1.41]. Spanish participants also had higher proportional reading time when compared to English participants, *p* = .029, 95% CI [.007, .189] and Polish participants, *p* = .015, 95% CI [.01, .20], i.e. the Spanish participants spent most time reading the subtitle while viewing the clip. Finally, Polish participants had a statistically lower mean fixation duration compared to English, *p* = .041, 95% CI [-38.10, -59], and Spanish, *p* = .003, 95% CI [-43.62, -7.16]. English and Spanish participants did not differ from each other in mean fixation duration, *p* =1.00, 95% CI [-23.55, 11.47].

**Table 13.** ANOVA results for between-subject effects in Experiment 1

Measure	df	*F*	*p*	𝜂_p_²
Absolute reading time	2,63	5.593	.006*	.151
Proportional reading time	2,63	5.398	.007*	.146
Mean fixation duration	2,63	6.166	.004*	.164
Fixation count	2,63	2.980	.058	.086
Revisits	2,63	.332	.719	.010

Overall, the results indicate that the processing of subtitles was least effortful for Polish participants and most effortful for Spanish participants.

### Experiment 2

A total of 46 participants (19 males, 27 females) took part in the experiment: 27 were hearing, 10 hard of hearing, and 9 deaf.

### Cognitive load 

We conducted 2 x 3 mixed ANOVAs on each indicator of cognitive load with segmentation (SS vs. NSS) as a within-subject variable and degree of hearing loss (hearing, hard of hearing, deaf) as a between-subject variable.

Similarly to Experiment 1, we found a significant main effect of segmentation on difficulty, effort, and frustration (Table 14). The NSS subtitles induced higher cognitive load than the SS condition in all groups of participants. There were no interactions.

**Table 14.** Mean cognitive load indicators for different participant groups in Experiment 2

	Degree of hearing loss						
	Hearing	Hard of hearing	Deaf	df	*F*	*p*	𝜂_p_²
	M (SD)	M (SD)	M (SD)				
Difficulty				1,43	6,580	.014*	.133
SS	2.37 (1.27)	1.60 (1.07)	2.56 (1.42)				
NSS	2.63 (1.44)	2.20 (1.31)	3.44 (1.59)				
Effort				1,43	4,372	.042*	.092
SS	2.78 (1.55)	1.60 (1.07)	2.78 (1.64)				
NSS	2.89 (1.60)	2.50 (1.35)	3.44 (1.42)				
Frustration				1,43	7,669	.008*	.151
SS	2.15 (1.40)	1.00 (.00)	2.56 (1.59)				
NSS	3.04 (1.85)	2.10 (1.28)	3.00 (1.58)				

There was no main effect of hearing loss on difficulty, *F*(2,43) = 2.100, *p* = .135, 𝜂_p_² = .089 or on effort, *F*(2,43) = 1.932, *p* = .157, 𝜂_p_² = .082, but there was an effect near to significance on frustration, *F*(2,43) = 3.100, *p* = .052, 𝜂_p_² = .129. Post-hoc tests showed a result approaching significance: hard of hearing participants reported lower frustration levels than hearing participants, *p* = .079, 95% CI [-2.17, .09]. In general, the lowest cognitive load was reported by hard of hearing participants.

### Comprehension 

Expecting that non-syntactic segmentation would negatively affect comprehension, we conducted a 2 x 3 mixed ANOVA on segmentation (SS vs. NSS) and degree of hearing loss (hearing, hard of hearing, and deaf).

**Table 15.** Descriptive statistics for comprehension in Experiment 2

	Deafness	Mean (SD)
Comprehension SS	Hearing	4.11 (1.01))
	Hard of hearing	4.60 (.51)
	Deaf	4.00 (.70)
	Total	4.20 (.88)
Comprehension NSS	Hearing	4.26 (1.02)
	Hard of hearing	4.50 (.70)
	Deaf	3.44 (1.23)
	Total	4.15 (1.05)

Note*:* Maximum score was 5

Despite our predictions, and similarly to Experiment 1, we found no main effect of segmentation on comprehension *F*(1,43) = .713, *p* = .403, 𝜂_p_² = .016. There were no interactions.

As for between-subject effects, we found a marginally significant main effect of hearing loss on comprehension, *F*(2,43) = 3.061, *p* = .057, 𝜂_p_² = .125. The highest comprehension scores were obtained by hard of hearing participants and the lowest by deaf participants (Table 15). Post-hoc analyses with Bonferroni correction showed that deaf participants differed from hard of hearing participants, *p* = .053, 95% CI [-1.66, .01].

### Eye tracking measures 

Due to problems with calibration, 10 participants had to be excluded from eye tracking analyses, leaving a total of 22 hearing, 8 hard of hearing, and 6 deaf participants.

To examine whether the non-syntactically segmented text resulted in longer reading times, more revisits and higher mean fixation duration, we conducted an analogous mixed ANOVA. We found no main effect of segmentation on any of the eye tracking measures (Table 16), but a few interactions between segmentation and deafness: in absolute reading time, *F*(2,33) = 4,205, *p* = .024, 𝜂_p_²= .203; proportional reading time, *F*(2,33) = 4,912, *p* = .014, 𝜂_p_² = .229; fixation count, *F*(2,33) = 3,992, *p* = .028, 𝜂_p_² = .195; and revisits, *F*(2,33) = 6,572, *p* = .004, 𝜂_p_² = .285.

**Table 16.** Mean eye tracking measures by segmentation in Experiment 2

	Hearing loss						
	Hearing	Hard of hearing	Deaf	Df	*F*	*p*	𝜂_p_²
Absolute reading time (ms)				1,33	1.752	.195	.050
SS	1614	1619	1222				
NSS	1617	1519	1522				
Proportional reading time				1,33	2.270	.141	.064
SS	.65	.66	.45				
NSS	.66	.61	.62				
Mean fixation duration (ms)				1,33	.199	.659	.006
SS	209	199	214				
NSS	211	185	219				
Fixation count				1,33	2.686	.111	.075
SS	6.41	6.73	4.63				
NSS	6.45	6.45	5.90				
Revisits				1,33	.352	.557	.011
SS	.28	.20	.45				
NSS	39	.30	.15				

We broke down the interactions with simpleeffects analyses by means of post-hoc tests using Bonferroni correction. In the deaf group, we found an effect of segmentation on revisits approaching significance, *F*(1,5) = 5.934, *p* = .059, 𝜂_p_² = .543. Deaf participants had more revisits in the SS condition than in the NSS one, *p* = .059. They also had a higher absolute reading time, proportional reading time, and fixation count in the NSS compared to the SS condition, but possibly owing to the small sample size, these differences did not reach statistical significance. In the hard of hearing group, there was no significant main effect of segmentation on any of the eye tracking measures (*ps*> .05). In the hearing group, there was no statistically significant main effect of segmentation (all *ps*> .05).

A between-subject analysis showed a close to significant main effect of degree of hearing loss on fixation count, *F*(2,33) = 3.204, *p* = .054, 𝜂_p_² = .163. Deaf participants had fewer fixations per subtitle compared to hard of hearing, *p* = .088, 95% CI [2.79, .14], or hearing participants, *p* = .076, 95% CI [-2.41, .08]. No other measures were significant.

### Interviews 

Following the eye tracking tests, we conducted short semi-structured interviews to elicit participants’ views on subtitle segmentation, complementing the quantitative part of the study (5). We used inductive coding to identify themes reported by participants. Several Spanish, Polish, and deaf participants said that keeping units of meaning together contributed to the readability of subtitles because by creating false expectations (i.e. “garden path” sentences), NSS line-breaks can require more effort to process. These participants believed that chunking text by phrases according to “natural thoughts” allowed subtitles to be read quickly. In contrast, other participants said that NSS subtitles gave them a sense of continuity in reading the subtitles. A third theme in relation to dealing with SS and NSS subtitles was that participants adapted their reading strategies to different types of line-breaks. Finally, a number of people also admitted they had not noticed any differences in the subtitle segmentation between the clips, saying they had never paid any attention to subtitle segmentation.

## Discussion 

The two experiments reported in this paper examined the impact of text segmentation in subtitles on cognitive load and reading performance. We also investigated whether viewers’ linguistic background (native language and hearing status) impacts on how they process syntactically and nonsyntactically segmented subtitles. Drawing on the large body of literature on text segmentation in subtitling (12, 18, 44, 45, 48) and literature on parsing and text chunking during reading (22, 23, 33, 39, 40, 51), we predicted that subtitle reading would be adversely affected by non-syntactic segmentation.

This prediction was partly upheld. One of the most important findings of this study is that participants reported higher cognitive load in nonsyntactically segmented (NSS) subtitles compared to syntactically segmented (SS) ones. In both experiments, mental effort, difficulty, and frustration were reported as higher in the NSS condition. A possible explanation of this finding may be that NSS text increases extraneous load, i.e. the type of cognitive load related to the way information is presented (58). Given the limitations of working memory capacity (4, 8), NSS may leave less capacity to process the remaining visual, auditory, and textual information. This, in turn, would increase their frustration, make them expend more effort and lead them to perceive the task as more difficult.

Although cognitive load was found to be consistently higher in the NSS condition across the board in all participant groups, the mean differences between the two conditions do not differ substantially and thus the effect sizes are not large. We believe the small effect size may stem from the fact that the clips used in this study were quite short. As cognitive fatigue increases with the length of the task, and declines simultaneously in performance (1, 53, 64), we might expect that in longer clips with non-syntactically segmented subtitles, the cognitive load would accumulate over time, resulting in more prominent mean differences between the two conditions. We acknowledge that the short duration of clips, necessitated by the length of the entire experiment, is an important limitation of this study. However, a number of previous studies on subtitling have also used very short clips (19, 20, 48, 52). In this study, we only examined text segmentation within a single subtitle; further research should also explore the effects of non-syntactic segmentation across two or more consecutive subtitles, where the impact of NSS subtitles on cognitive load may be even higher.

Despite the higher cognitive load and contrary to our predictions, we found no evidence that subtitles which are not segmented in accordance with professional standards result in lower comprehension. Participants coped well in both conditions, achieving similar comprehension scores regardless of segmentation. This finding is in line with the results reported by Perego et al. (46), using Italian participants, that subtitles containing non-syntactically segmented noun phrases did not negatively affect participants’ comprehension. Our research extends these findings to other linguistic units in English (verb phrases and conjunctions as well as noun phrases) and other groups of participants (hearing English, Polish, and Spanish speakers, as well as deaf and hard of hearing participants). The finding that performance in processing NSS text is not negatively affected despite the participants’ extra effort (as shown by increased cognitive load) may be attributed to the short duration of the clips and also to overall high comprehension scores. As the clips were short, there were limited points that could be included in the comprehension questions. Other likely reasons for the lack of significant differences between the two conditions is the extensive experience that all the participants had of using subtitles in the UK, and that participants may have become accustomed to subtitling not adhering to professional segmentation standards. Our sample of participants was also relatively well-educated, which may have been a reason for their comprehension scores being near ceiling. Furthermore, as noted by Mitchell (40), when interpreting the syntactic structure of sentences in reading, people use non-lexical cues such as text layout or punctuation as parsing aids, although these cues are of secondary importance when compared to words, which constitute “the central source of information” (p. 123). This is also consistent with what the participants in our study reported in the interviews. For example, one deaf participant said: “Line breaks have their value, yet when you are reading fast, most of the time it becomes less relevant.”

In addition to understanding the effects of segmentation on subtitle processing, this study also found interesting results relating to differences in subtitle processing between the different groups of viewers. In Experiment 1, Spanish participants had the highest cognitive load and lowest comprehension, and spent more time reading subtitles than Polish and English participants. Although it is impossible to attribute these findings unequivocally to Spanish participants coming from a dubbing country, this finding may related to their experience of having grown up exposed more to dubbing than subtitling. In Experiment 2, we found that subtitle processing was the least effortful for the hard of hearing group: they reported the lowest cognitive effort and had the highest comprehension score. This result may be attributed to their high familiarity with subtitling (as declared in the pre-test questionnaire) compared to the hearing group. Although no data were obtained for the groups in Experiment 2 in relation to English literacy measures, as a group, individuals born deaf or deafened early in life have low average reading ages, and more effortful processing by the deaf group may be related to lower literacy.

Different viewers adopt different strategies to cope with reading NSS subtitles. In the case of hearing participants, there were more revisits to the subtitle area for NSS subtitles, which is a likely indication of parsing difficulties (51). In the group of participants with hearing loss, deaf people spent more time reading NSS subtitles than SS ones. Given that longer reading time may indicate difficulty in extracting information (17), this may also be taken to reflect parsing problems. This interpretation is also in accordance with the longer durations of fixations in the deaf group, which is another indicator of processing difficulties (17, 49). Unlike the findings of other studies (26, 60, 61), in this study, deaf participants fixated less on the subtitles than hard of hearing and hearing participants. Our results, however, are in line with a recent eye tracking study (38), where deaf people also had fewer fixations than relation hearing viewers. According to Miquel Iriarte (38), deaf viewers relate to the visual information on the screen as a whole to a greater extent than hearing viewers, reading the subtitles faster to give them more time to direct their attention towards the visual narrative.

## Conclusions 

Our study has shown that text segmentation influences the processing of subtitled videos: nonsyntactically segmented subtitles may increase viewers’ cognitive load and eye movements. This was particularly noticeable for Spanish and deaf people. In order to enhance the viewing experience, using syntactic segmentation in subtitles may facilitate the process of reading subtitles, thus giving viewers greater time to follow the visual narrative of the film. Further research is necessary to disentangle the impact of the viewers’ country of origin, familiarity with subtitling, reading skills, and language proficiency on subtitle processing.

This study also provides support for the need to base subtitling guidelines on research evidence, particularly in view of the tremendous expansion of subtitling across different media and formats. The results are directly applicable to current practices in television broadcasting and video-on-demand services. They can also be adopted in subtitle personalization to improve automation algorithms for subtitle display in order to facilitate the processing of subtitles among the myriad different viewers using subtitles.

## Ethics and Conflict of Interest 

The author(s) declare(s) that the contents of the article are in agreement with the ethics described in http://biblio.unibe.ch/portale/elibrary/BOP/jemr/ethics.html and that there is no conflict of interest regarding the publication of this paper.

## Acknowledgements

The research reported here has been supported by a grant from the European Union’s Horizon 2020 research and innovation programme under the Marie Skłodowska-Curie Grant Agreement No. 702606, “La Caixa” Foundation (E-08-2014-1306365) and Transmedia Catalonia Research Group (2017SGR113).

Many thanks for Pilar Orero and Gert Vercauteren for their comments on an earlier version of the manuscript.
